# Revised phylogeny of mouflon based on expanded sampling of mitogenomes

**DOI:** 10.1371/journal.pone.0323354

**Published:** 2025-05-14

**Authors:** Paolo Mereu, Monica Pirastru, Pedro Morell Miranda, Gözde Atağ, Kıvılcım Başak Vural, Barbara Wilkens, André Elias Rodrigues Soares, Damla Kaptan, Marco Zedda, Nicolò Columbano, Mario Barbato, Salvatore Naitana, Eleftherios Hadjisterkotis, Mehmet Somel, Füsun Özer, Torsten Günther, Giovanni Giuseppe Leoni

**Affiliations:** 1 Dipartimento di Scienze Biomediche, Università degli Studi di Sassari, Sassari, Italy; 2 Human Evolution Program, Institute for Organismal Biology, Uppsala University, Uppsala, Sweden; 3 Population Genomics Group, Department of Veterinary Sciences, Ludwig-Maximilian University of Munich, Munich, Germany; 4 Department of Biological Sciences, Biology/Molecular Biology and Genetics, Middle East Technical University, Ankara, Turkey; 5 Independent Archaeologist, Alghero, Italy; 6 Dipartimento di Medicina Veterinaria, Università degli Studi di Sassari, Sassari, Italy; 7 Department of Veterinary Sciences, Università degli Studi di Messina, Messina, Italy; 8 Agricultural Research Institute, Ministry of Agriculture, Rural Development and Environment, Nicosia, Cyprus; 9 Department of Anthropology, Faculty of Letters, Hacettepe University, Ankara, Turkey; 10 Department of Social Anthropology, Hacettepe University, Ankara, Turkey; Institut des Regions Arides de Medenine: Institut des Regions Arides, TUNISIA

## Abstract

Mouflons are flagship species of the Mediterranean islands where they persist. Once thought to be the remnants of a European wild sheep population, archaeology suggests they were introduced by humans to the islands of Cyprus in the Early Neolithic (~10,000 years ago) and later to Corsica and Sardinia. Their status as truly wild animals remains a subject of debate. To investigate the phylogenetic relationship between these island populations and other domestic and wild sheep from the Mediterranean region, we sequenced 50 mitogenomes of mouflons from Sardinia and Corsica, and modern and ancient Sardinian domestic sheep. A total of 68 additional publicly available mitogenomes were included in the comparative analysis and used to reconstruct the phylogeny of sheep and its closest wild relative, the mouflon (*Ovis gmelini*). Our study analyzed the evolutionary relationships within the C-E-X and haplogroup B clusters, showing that: a) Cyprus mouflons are more related to Anatolian and Iranian mouflons belonging to the wild haplogroup X, which seems to be basal to the domestic C and E haplogroups; b) Corsican and Sardinian mouflon arise from basal lineages associated with the early European expansion of domestic sheep. These results highlight the phylogenetic distinctiveness of the mouflon populations from the Mediterranean islands, suggesting a revision of their systematic classification and an update of the nomenclature for Sardinian and Corsican mouflons from the current status of subspecies of domestic sheep (*Ovis aries musimon*) to subspecies of their wild relatives (*Ovis gmelini musimon*) which would facilitate conservation efforts.

## Introduction

Based on bone remains found in early Neolithic settlements, the process of domestication of sheep (*Ovis aries*) from the Asiatic mouflon (*Ovis gmelini*), started 10,000–12,000 years ago in the Fertile Crescent. Since no such remains were found in Europe, it was assumed that this continent was not inhabited by wild sheep during that time [1–6]. To a certain extent, this would explain the early expanding success of domestic sheep as livestock. While pigs, cattle and goats competed and, in some cases, admixed with their wild counterparts in their new environment [7–9], sheep invaded unexploited habitats. These early sheep, however, were quite different from their current phenotype. Following domestication, they have gradually changed from their Asiatic mouflon ancestors with horns in both sexes, coarse hair and a specific color pattern to the mostly polled, woolly and white sheep breeds we find today [6,10]. There are exceptions, however, as several islands allowed for more archaic phenotypes to survive to the present, such as the Soay sheep from the island of St. Kilda in Scotland [11].

While the current native range of the ancestral Asiatic mouflon extends from eastern Turkey, Armenia, southern Azerbaijan to Iran, the scientific denomination of the mouflons occurring on Mediterranean islands (Cyprus, Corsica and Sardinia), and subsequently introduced into continental Europe during the 18^th^ century [12,13], is highly controversial [14–17].

Both the Corsico-Sardinian and the populations translocated to continental Europe are commonly referred to as “European mouflon”. The term ‘mouflon’ was broadly used by some authorities to refer to close relatives of domestic sheep living in the wild [19]. However, archaeological works [15,20,21] and genetic studies [13,16,22–27] as well as phenotypic observations [28] provided sufficient evidence to rank the European mouflon as subspecies of the Asiatic one [13,29].

The island mouflon populations of Corsica and Sardinia have experienced a considerable population and range reduction. In addition, they have retreated into isolated mountainous regions of their respective islands, forming several discrete populations [4,26,30]. Therefore, local wildlife authorities started to implement a number of management and conservation programs [26,31,32]. As a consequence of these management efforts, Sardinian mouflons have been reintroduced successfully into some new regions of the island. Mouflons in Cyprus are only found in the mountainous Paphos forest in western Cyprus [27,33,34].

Whole genome sequencing has been conducted on European mouflons [35–37]; however, the sampled individuals were sourced from outside the islands. As a result, these samples cannot provide information on the original population structure of Corsica and Sardinia and may only reflect a subset of their genetic diversity. A recent whole-genome study suggested that the Cyprus mouflon is closely related to European mouflons and domestic sheep [27]. These studies have primarily focused on analyzing demographic history to describe continental relationships, with a particular emphasis on domestic sheep.

To investigate genetic diversity and population structure, genome-wide SNP array studies were conducted with mouflon populations originating from the islands [3,38], but, again, these studies focused on the relationship between domestic breeds and the different mouflon populations. Other authors used microsatellites and partial mitogenomes (mainly Cytochrome B and control regions) [4,26,39,40] to describe genetic structure and evolutionary relationships of the islands populations. A study focusing on Sardinia reported over the occurrence of three sub-populations, with Montes Forest putatively harboring the ancestral haplotype of Sardinian mouflons [4,25]. A similar study described two geographically separated sub-populations of Corsica with a clear genetic isolation and modelled a single introduction event plus long-time isolation as the most likely explanation for the observed pattern [26]. Regarding Cyprus mouflon, several mitogenomic studies suggested they are genetically closer to Near Eastern mouflon such as the Anatolian and Iranian mouflon (*O. gmelini*) than to those from Corsica and Sardinia [23,39]. More recently, Guerrini et al. [41] proposed renaming Haplogroup (HPG) X, originally reported in Anatolian mouflon [22], as HPG-C2, given that all sampled Cyprus mouflons clustered with them and formed a sister group to HPG-C. While these studies are informative of the broader patterns of the Mediterranean mouflon populations, the use of short mitochondrial DNA (mtDNA) fragments limit the resolution needed to describe fine-scale relationship within the species [42]. A significant advancement came with the recent sequencing of the complete mitogenome of a Sardinian mouflon which allowed for a redefinition of the phylogeny of HPG-B. This study placed Sardinian mouflons as basal to all other sampled domestic or feral sheep from this haplogroup [25].

In the present study, the phylogenetic relationship between Mediterranean mouflon populations, and between them and other continental mouflon populations were investigated by complete mitogenomes analysis. The aim was to clarify the evolutionary origin of each Mediterranean mouflon population and their phylogenetic relationships with both their wild relatives from Continental Europe and Near East and domestic sheep. Bridging this knowledge gap is essential for refining our understanding of their taxonomy and potentially resolving the long-standing classification issue. Currently, the Corsico-Sardinian mouflon is classified as a subspecies of domestic sheep, whereas the Cyprus mouflon has recently been recognized as a wild species due to its direct descent from the Asian mouflon. By providing a more comprehensive evolutionary framework, this study seeks to contribute to a more accurate and scientific evidence-based classification of these populations. In order to achieve this goal, we sequenced the whole genomes and assembled the complete mitogenome of 50 *Ovis* samples including 35 mouflons from the Mediterranean islands of Sardinia (n = 21) and Corsica (n = 14), 12 domestic sheep with Sarda (n = 6) and Pecora Nera di Arbus (n = 6) breeds from Sardinia, and three ancient DNA samples from Sardinia (S1 Table). We then compared them with 25 newly assembled mouflon mitogenomes from Anatolian mouflons (n = 5) (*O. g. anatolica*), Cyprus mouflons (n = 10) (*O. g. ophion*) [27] and Iranian mouflons (n = 20) (*O. gmelini*) [43] (S1 Table). A set of modern and primitive sheep belonging to all known mitochondrial haplogroups with a main focus on European sheep were also included in the dataset along with six ancient samples from Iberia (S1 Table) [44]. Finally, seven additional sequences were included as outgroups to calibrate the molecular dating analysis (S1 Table). This wide sampling focused on the Mediterranean basin allowed us to describe the phylogenetic relationship between the different mouflon populations of the Mediterranean and the Middle East, with a particular focus on the mouflon from Corsica and Sardinia, whose lineages were found to belong to HPG-B [3,4,25,26,38,39], the most widespread haplogroup [45] and traditionally associated with the early Neolithic colonization of Europe [46–48].

## Materials and methods

### Ethics statement and authorizations

Blood samples from Sardinian and Corsican mouflons were collected in strict accordance with the guidelines of the Ethics Committee of Sassari University, Italy, and the cabinet vétérinaire Les Deux Iles Santa Maria Siché, Corse du Sud, France, respectively. The blood samples were taken by highly trained veterinary personnel without any anesthesia and did not cause any suffering or stress to the animals involved. There was no health risk to the animal. All necessary permits were obtained for the described study, which complied with all relevant regulations.

The ancient Sardinian samples analyzed in this study have been made available by Superintendency for archaeological heritage for the provinces of Sassari and Nuoro (Sardinia, Italy).

### DNA sampling, extraction and sequencing

**Fig 1:**
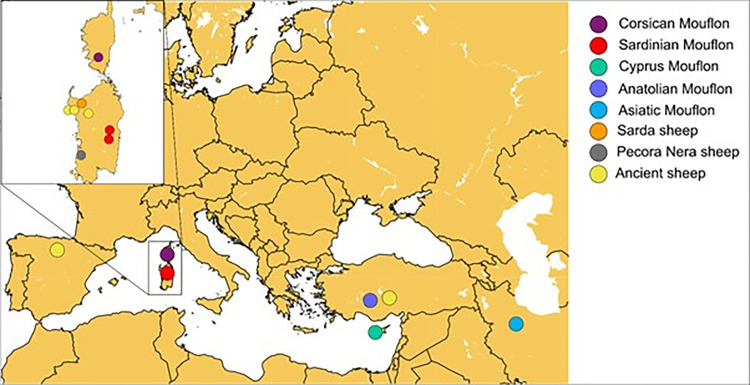
Distribution of the relevant modern domestic and wild and ancient domestic samples included in this study. Map generated using pyGMT [18].

The sampling areas of the mouflon and sheep samples included in this study is reported in Fig 1. Genetic material from all the modern samples was extracted from whole blood using the GenElute Mammalian Genomic DNA Miniprep Kit (Sigma- Aldrich, Missouri, USA) following the standard protocol. The Corsican mouflon samples were collected from the areas of Monte Cinto (42.383° N, 8.898° E) and the massif of Bavella (41.785° N, 9.266° E), respectively in the north-west and south-east of the island. The Sardinian mouflon samples were collected from two locations geographically included in the historical range [49] of the species: the forest of Montes in Barbagia (Orgosolo) (40.072° N, 09.235° E) and Mount Tonneri in Ogliastra (Lanusei) (39.534° N, 9230° E) (Fig 1). Sequencing libraries were prepared at the SciLifeLab SNP&SEQ Technology Platform at Uppsala University using the TrueSeq Nano kit with the exception of the Cor-B_m01 and Sar-T_m17 samples, for which a ThruPlex DNA-seq kit was used. All samples were sequenced as parts of Illumina NovaSeq 6000 S4 lanes with 150 bp paired end reads at the SciLifeLab SNP&SEQ Technology Platform at Uppsala University.

Ancient DNA from six samples from Sardinia was extracted [50] and amplified into double-stranded blunt-end libraries [51] at the dedicated ancient DNA facilities of the SciLifeLab ancient DNA unit in Uppsala, Sweden. Libraries were prepared using a MinElute PCR purification kit. Libraries were then sequenced in equimolar ratios and sequenced as part of a lane of an Illumina NovaSeq S4 flow cell using v1.5 chemistry and 150 bp paired end reads, at SciLifeLab National Genomics Infrastructure in Stockholm. Only three of the ancient samples, AALG-001M from the neolithic site of “Grotta Verde” (40°33’53.93” N - 08°09’54.18” E), AALG-003 from the prehistoric building “Nuraghe Santu Antine” in the Nuraghi Valley (40°29’10.9” N - 08°46’11.82” E) and AALG-004 from the Sacred Well “La Purissima” in the Archaeological Park of Santa Cristina (40°33’50” N -8°20’44” E) showed viable levels of DNA preservation for further analysis (endogenous DNA > 1.5%). In addition to these Sardinian samples, two ancient Anatolian samples previously sequenced by Yurtman and colleagues [47] were included into the dataset.

Iranian, Anatolian and Cyprus mouflons were not sequenced as part of this study. They were available from the literature [27,43] and FASTQ files were downloaded from the European Nucleotide Archive (ENA) (NextGen projects PRJEB7436, for Iranian mouflons, and PRJEB69690 for Anatolian and Cyprus mouflons) (S1 Table). Mitochondrial reads for the newly presented samples are available from ENA under accession PRJEB76447 (https://www.ebi.ac.uk/ena/browser/view/PRJEB76447) (S1 Table).

### Mitochondrial DNA sequences assembly

Mitochondrial consensus sequences were assembled from FASTQ files using the *Mapping Iterative Assembler (MIA)* [52], a reference-based iterative assembler using the reference sequence for the Iranian Mouflon (NCBI Reference Sequence NC-026063). Only sites with a minimum coverage of 10x, with mapping quality ≥40 and two-thirds base agreement on each position were considered for calling a consensus. All sites not meeting these requirements were set up as “N”. All sequences were then aligned and visually inspected using *MAFFT* [53]. Ancient sequences were aligned using a custom substitution matrix to take into account deamination damage. Haplogroups were inferred using *MitoToolsPy 1.0* [54] using the sheep reference and the whole mitogenome.

### Phylogenetic inference

Sequence alignment was carried out with *BioEdit 7.7.1* [55]. Firstly, sequences were aligned using the *ClustalW* algorithm [56], and then the alignment was reviewed and corrected manually, with a special focus on the tandem repeats of the D-Loop region. Once the sequences were aligned, *MEGA 11.0.13* [57] was used to find the best-fitting model (*GTR + G + I*). This model was used in *MrBayes 3.2.7* [58] to reconstruct the phylogeny through Monte Carlo Markov Chains (MCMC) to approximate the posterior probability of the trees. The first 25% of the samples were discarded in the burn-in step, and the MCMC was performed on six runs with 2,198,000 generations. The tree was visualized using *FigTree 1.4.4* (http://tree.bio.ed.ac.uk/). The branches of the plots were transformed using the *’proportional’* setting to increase their readability.

### Radiocarbon dating

Three novel ancient samples from Sardinia showed sufficient amounts of endogenous DNA and were radiocarbon dated at the Tandem Laboratory at Uppsala University (S2 Table). Teeth and bone from sub-samples were mechanically cleaned by scraping their surface and then grounded in a mortar. First, an incubation was performed in 0.25 M HCl for 48 hours at room temperature and then the soluble fraction was incubated in 0.01 M HCl at 50°C for 16 hours. The soluble fraction was added to a 30 kDa ultrafilter and centrifuged, and the retentate was lyophilized. Finally, the fraction to be dated was combusted to CO_2_ using a Fe-catalyst before determination. Raw dates were calibrated using *OxCal 4.4* [59] using *IntCal20* calibration curve [60].

### Molecular dating

Divergence time estimation was inferred by mitogenome sequence analysis using a Bayesian approach as implemented in *BEAST 1.10.4* [61]. Overall, a total of 10 calibration points (CPs) based on fossil records were used: four providing time estimates for nodes within Bovidae [62] and six - including the three fossil records radiocarbon dated in the present study - for nodes inside the Moufloniformes clade (S3 Table). Sequences were analyzed under the GTR + G + I model of sequence evolution, assuming 4 gamma categories, a Yule process speciation rate, and empirical base frequencies. We compared the two most used approaches to infer molecular dating: a) the lognormal relaxed clock with uncorrelated rates and b) the strict clock. A Bayes Factors comparison analysis implemented in *Tracer 1.7.2* [63] was performed to test the two clock models and the relaxed clock was found to be the most suitable for the used dataset. Two independent Markov chain Monte Carlo runs were carried out with 200,000,000 iterations each, and sampled every 10,000 steps after a 10% burn-in. The runs were combined after checking for convergence > 100. A maximum clade credibility (MCC) tree was generated using *TreeAnnotator 1.10.4* [61] and visualized with *FigTree 1.4.4* (http://tree.bio.ed.ac.uk/).

## Results

Consensus mtDNA genome sequences were successfully assembled for 35 mouflons from Corsica and Sardinia, along with those from six Sarda sheep and six Pecora Nera di Arbus. We also obtained consensus mtDNA genome sequences from three ancient Sardinian samples excavated from a Neolithic context that radiocarbon dating assigned to the Roman and Modern periods.

### Phylogeny

**Fig 2:**
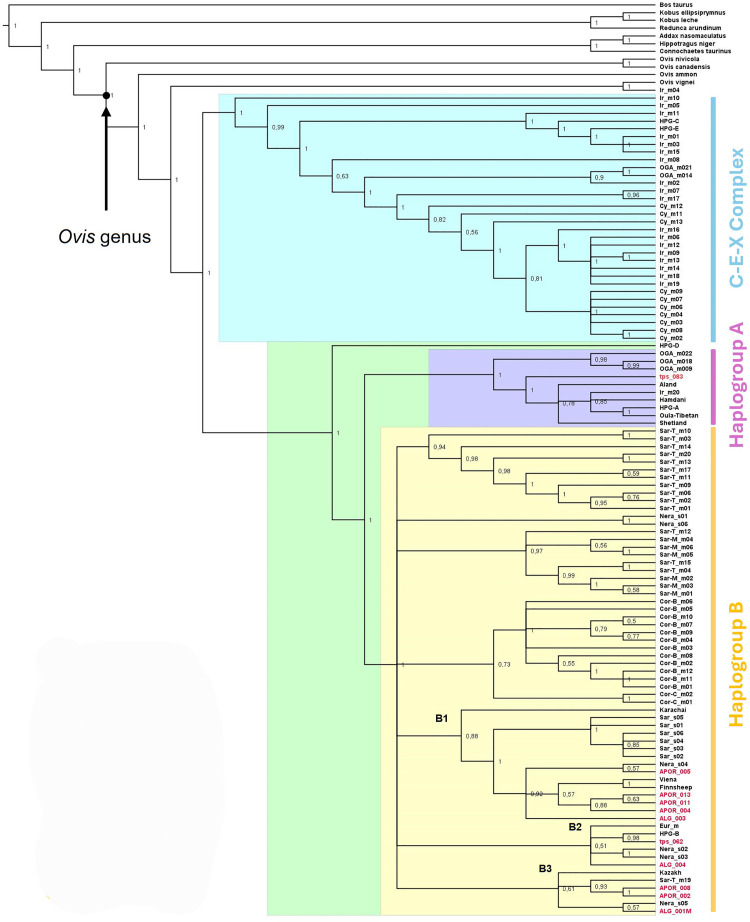
Bayesian phylogenetic reconstruction of wild and domestic sheep.

We reconstructed the phylogeny of the Mediterranean mouflons employing a Bayesian phylogenetic inference (BI) approach using whole mitogenomes. The resulting tree placed all the Corsican and Sardinian mouflons as part of the HPG-B cluster (Fig 2).

The Corsican cluster was composed of two sub-groups, one encompassing the two samples from the Cinto area (labelled “Cor-C” in Fig 2), located in the north of the island, and the other grouping all samples from Bavella (Cor-B), in the south. This separation aligns with previous analyses highlighting strong genetic differentiation between the two populations [26].

The Sardinian mouflons were grouped in two clusters; one including exclusively mouflons from the South-Western population (labelled “Sar-T” in Fig 2) and the other composed of a mix of North-Eastern (“Sar-M”) and South-Western individuals. The other four lineages within HPG-B belonged to a mix of domestic sheep from Europe and Central Asia, plus two Pecora Nera di Arbus individuals that cluster together. These subclusters in HPG-B (labelled B1, B2 and B3 in Fig 2), with the exception of the one grouping exclusively Pecora Nera di Arbus individuals, also included all ancient mitogenomes from Iberia and Sardinia plus one of the Anatolian Neolithic sheep. We also detected the presence of Sarda sheep and Pecora Nera di Arbus and one Sardinian mouflon within these domestic clusters, with all Sarda individuals forming their own monophyletic group.

Within the HPG-A cluster we detected a group of Anatolian mouflon individuals, basal to domestic samples from this haplogroup. Further, we found a Neolithic Anatolian domestic sheep as a basal branch from more modern domestic samples and a wild Iranian mouflon (Ir_m20).

Two of the Anatolian mouflons were included into the C-E-X complex, which also incorporated two domestic sequences used to define the HPG-C and HPG-E, respectively, most of the Iranian Mouflons and all the individuals from Cyprus. All other wild individuals on the C-E-X complex fell within a sister clade to this C-E cluster, with one Iranian mouflon as basal, followed by the two Anatolian mouflons belonging to HPG-X - also referred to as C2 by Guerrini et al. [41] - and one Iranian individual. As a sister group of this trio, we found all the Cyprus mouflon samples along with two clusters of Iranian mouflons.

### Molecular dating

The divergence times of the main splitting events within the genus *Ovis*, with a particular focus on the Moufloniformes group, resulting from multiple calibration points (see S3 Table for further details), are reported in Table 1.

**Table 1:** Divergence time estimates in millions of years of the main splitting events within *Ovis* as inferred through mitogenome analysis. Median divergence and 95% height posterior density (HPD) are provided.

	MEDIAN	95% HPD
***Ovis***	4.615	1.968 - 9.439
***Canadensis/Nivicola split***	1.589	0.684 - 3.28
***Argali lineage***	2.221	0.936 - 4.532
***Urial lineage***	1.794	0.751 - 3.657
***Mouflon&sheep group***	0.810	0.338 - 1.655
***C-E-X complex***	0.518	0.217 - 1.063
***HPG-C/ HPG-E split***	0.245	0.101 - 0.506
***HPGs C-E/ C-E-X mouflon split***	0.387	0.161 - 0.792
***HPG-D/ HPGs A - B split***	0.669	0.279 - 1.369
***HPG-A/ HPG-B split***	0.531	0.224 - 1.092
***HPG-A***	0.156	0.061 - 0.328
***HPG-A mouflon***	0.091	0.033 - 0.199
***HPG-A sheep***	0.093	0.035 - 0.196
***HPG-B***	0.162	0.065 - 0.338
***Corsican mouflon***	0.085	0.029 - 0.192
***Sardinian mouflon (Ogliastra)***	0.084	0.032 - 0.178
***Sardinian mouflon (Montes)***	0.118	0.047 - 0.247
***Sub-clade B1 (Karachai)***	0.118	0.047 - 0.248
***Sarda sheep/ APOR-ALG-Nera-primitive split***	0.099	0.039 - 0.207
***Sarda sheep***	0.055	0.019 - 0.119
***APOR-ALG-Nera-primitive sheep***	0.083	0.033 - 0.175
***Sub-calde B2 (EurM)***	0.078	0.029 - 0.167
***Sub-clade B3 (Kazakh)***	0.105	0.042 - 0.219

Based on the relaxed clock results, *Ovis* species showed up about 4.6 MYA, while argali and urial split 2.2 and 1.8 MYA, respectively, from Moufloniformes which shared a common ancestor that lived about 810 KYA.

The split between the mtDNA lineages leading to HPG-D and to the cluster including HPG-A and HPG-B occurred 670 KYA. Later, the separation between HPG-A and HPG-B lineages occurred ca. 531 KYA. Within the HPG-A, the Anatolian mouflon lineage stemmed approximately 91 KYA from a group including mostly primitive sheep, such as Aland, Shetland, Hamdani and Oula Tibetan sheep, with the tps_083 sequence from a Neolithic Anatolian domestic sheep sample representing a basal lineage which early split from the other at around 90 KYA. The most recent common ancestor (MRCA) of the HPG-A lived about 150 KYA. The HPG-B radiation started around 160 KYA. The appearance of the Corsican mouflon clade dates back to 85 KYA. The rise of the two clades, one exclusive to the Sardinian mouflons from Ogliastra and the other including the mixed group from the Ogliastra and Montes Forest areas, occurred 84 KYA and 118 KYA, respectively. Within the sub-clade B1 the split of the Karachai sheep lineage was estimated at ca. 118 KYA, while the Sarda sheep lineage diverged from the other species 99 KYA. The most common recent ancestor of the sub-clades B2 and B3 lived 78 KYA and 105 KYA, respectively.

The radiation of the lineages within the C-E-X complex started at 518 KYA. Approximately 387 KYA, a divergence occurred within this cluster which separated a group containing domestic sheep (defining HPGs C and E) and four Iranian mouflon sequences from another group consisting of Anatolian, Cyprus, and most Iranian mouflon sequences (excluding Ir_m10 and Ir_m05). The early lineages radiation within these two clades took place around 340–350 KYA. The group composed of OGA_m014, OGA_m021 and Ir_m02 sequences shared a MRCA who lived around 158 KYA.

## Discussion

In this study, the largest dataset of complete mitogenomes from Corsican and Sardinian mouflon so far available was generated. We provided a mitogenome-based phylogeny of the *Ovis* genus and the divergence time estimation of the main splitting events characterising the adaptive radiation of Moufloniformes. Damage patterns analysis of the ancient samples used to calibrate phylogenetic reconstruction showed a typical deamination pattern manifested in an increase of transition substitutions at the ends of the sequences and overall short fragments (S1 Fig). According to the guidelines reported in Briggs et al. [64], this evidence allowed us to validate the ancient origin of the sequence data. Our results highlight the complex phylogeographic history of wild, domestic and feral sheep in Western Eurasia. All the mitogenome sequences from domestic sheep individuals grouped within the *O. gmelini* clade, in accordance with the hypothesis that the current domestic sheep breeds descended from ancestral evolutionary lineages of the Asiatic mouflon [6,22,39,48]. Each mouflon population shows their own and unique pattern, highlighting their different evolutionary histories, in relation to their geographical isolation. According to previous studies [3,4,25,26,38,39], all Corsican and Sardinian mouflons clustered within HPG-B (Fig 2). In addition to its predominance in Eurasia [45], HPG-B has been suggested to be the one harbored by the first Neolithic founders who moved into Europe from Near East [46–48].

The phylogenetic tree topology could not resolve the split between mouflons and domestic sheep in HPG-B, despite the previously reported basal position of one Sardinian mouflon from Montes Forest to all other HPG-B domestics and feral sheep [25]. The clade of Sardinian mouflon composed of a mix of Sar-M and Sar-T individuals (see Fig 2 for further details) supports a weak connectivity between Montes Forest and Mount Tonneri that still persist despite the presence of artificial barriers limiting individual’s dispersion [4]. All the ancient mitogenomes from Iberia and Sardinia plus one of the Anatolian Neolithic sheep grouped within domestic sheep sub-clade (Fig 2), but they do not cluster with each other either by region or period. The commercially bred and extremely popular Sarda sheep forms a tightly related cluster. Conversely, although belonging to a small, locally bred population of archaic sheep, the Pecora Nera di Arbus seems to harbor several different lineages [65]. The observation that ancient mitogenomes from Anatolia, Sardinia and Iberia cluster together with modern sheep both from the island and other regions of Eurasia, suggests that these lineages might precede the Neolithic expansion.

As reported in previous studies [22], Anatolian mouflon split into two haplogroups, one of which corresponds with a basal lineage of HPG-A. The basal position of the HPG-A Anatolian mouflons to a Neolithic Anatolian domestic sheep (tps_083), may point to a role of these Anatolian mouflons’ lineage during sheep domestication. Furthermore, we detected with HPG-A a wild Iranian mouflon (Ir_m20) for which signs of domestic admixture have been previously described [66] within the modern domestic branches.

The other Anatolian mouflon cluster corresponds to HPG-X [22]. Based on control region analyses, Guerrini and colleagues [41] suggested changing the name of HPG-X to HPG-C2, as they found HPG-X stemming from HPG-C. In the same study [41] it has been suggested that the HPG-X cluster is basal to all Cyprus mouflons. Our mitogenome-based results showed a more complex scenario, where the Cyprus mouflons were found to be more closely related to Iranian mouflons than to Anatolian mouflons, as previously proposed by Guerrini et al. [41]. This discrepancy is presumably due to the absence of Iranian samples in the paper of Guerrini et al. [41]. A recent study using whole-genome sequencing placed the Cyprus mouflons as a closer relative to the Anatolian population [27], something which agrees with the phenotypic observations [28,29,67] and with the results by Guerrini et al. [41]. Such a discrepancy with our results can be explained by considering the different peculiarities of these two markers. Although recent studies report that mtDNA may in rare cases undergo recombination and be under selection [68–71] it is generally assumed to be clonally inherited and to evolve under a near neutral selection [72–74]. For this reason, it is considered to be an ideal marker for phylogenetic inferences and accurate dating of samples [73,75]. Given that, events like different effective population size, incomplete lineage sorting and sex-biased dispersal can impact on mtDNA susceptibility to genetic drift leading to discordant results with nuclear DNA (nuDNA) [76,77]. As a consequence, when a large population hosting a high variability is fragmented into small isolates, mtDNA will diverge while the nuDNA will retain variability [76]. Accordingly, the fragmentation of the early population in pre-pottery Neolithic Anatolia, showing a high haplogroup variability [78] into small, isolated groups, could have led to the differential fixation of mtDNA haplotypes in the geographical isolates as in the case of the Cyprus mouflons.

Within the C-E-X complex, two Iranian mouflon samples seemed to be basal to the C-E-X complex, possibly representing a wild ancestral lineage. While the C-E complex only has two domestic sheep in our analysis due to our focus on the Mediterranean region, three wild Iranian mouflons cluster within HPG-E, which may suggest the occurrence of crossbreeding events between mouflon and domestic sheep.

### Molecular dating and phylogenetic distinctiveness

Based on our results, the early radiation of the genus *Ovis* occurred 4.6 MYA, which predates that of 3.8 proposed by Ropiquet and Hassanin [79], an estimate inferred from the analysis of the concatenated sequence of two mitochondrial and one nuclear genes. Our molecular estimate falls exactly within the range 2.2–5.4, according to previous studies [80,81] which support the origin of the genus *Ovis* in the Early Pliocene. The next split is the separation of the argali lineage (2.2 MYA), which is consistent with the estimates obtained by Meadows et al. [82] and Yang et al. [83], followed by the divergence between *O. nivicola* and *O. canadensis* dated back at 1.6 MYA, as previously reported [16]. The MRCA of mouflons and sheep dates back to 810 kya, which is consistent with what previously proposed by Meadows et al. [82] on the basis of mtDNA protein coding sequences, and 250 KY earlier than suggested by Portanier et al. [26] based on the CytB sequences analysis. The early radiation of mouflon and sheep was characterized by the divergence of two evolutionary lineages that gave rise to two clusters of mitochondrial haplogroups corresponding to those described by Demirci et al. [22]: cluster I, including HPGs A, B and D of the domestic sheep, and cluster II, referred to in our phylogenetic tree as the C-E-X Complex (Fig 2).

The mouflon lineages belonging to HPG-B (i.e., Sardinian and Corsican mouflon) originated in a time span ranging from 120 to 80 KYA and evolved separately from those identifying domestic sheep breeds, as shown by the tree topology. The only exception to this observation is represented by the position of the Sardinian mouflon named “SarT-m19”, which does not cluster with the other Sardinian mouflons, consistent with the occurrence of occasional crossbreeding between wild mouflon and domestic sheep [3,84]. Overall, such a phylogenetic distinctiveness with a clear differentiation between wild and domestic lineages can be seen in light of what was suggested by Chessa et al. [2] regarding the hypothesis of the two waves of ovine expansion, the first one involving mouflon-like animals, with horns and short-shedding wool, and the second one, occurred about 6000 years later, relating to the woolly individuals, that led to the modern sheep. It is assumed that the “domestication” of the first individuals was limited to protection against predators and to having a constant and readily available source of meat and skins, with only few interactions between animals and humans and no artificial selection [1]. As a consequence of the second wave, the ancestral livestock were freed in favor of the new economically valuable one with the consequent return to the wild. The Sardinian and Corsican mouflons would therefore be remnants of those early domestic ancestors, which proliferated undisturbed under a reduced level of competition due to both geographic and genetic isolation [6,26]. Even within the HPG-A cluster, the evolutionary lineage of the wild Anatolian mouflon (*O. g. anatolica*) split from that of the domestic breeds that cluster with the ancient Anatolian sample tps_083. This separation event predates the sheep domestication process (Table 1), as observed for the wild lineages within the HPG-B cluster. Evolutionary distinctiveness of mouflon and domestic sheep is also highlighted in the C-E-X complex, with the exception of samples Ir_m11, Ir_m01, Ir_m03 and Ir_m15 which show ancestry from domestic ewes.

### Mouflon origin, systematic and nomenclature

Overall, these results raise an issue that deserves in-depth discussion, namely the nomenclature of taxa grouped in the Moufloniformes cluster, whose taxonomic classification remains controversial and a source of confusion. We suggest revising the nomenclature as it follows. Given that *O. gmelini* is now considered to be the most credited ancestor of the extant mouflon (as agreed by all the wild sheep experts during the 6^th^ World Congress on Mountain Ungulates and the 5^th^ International Symposium on Mouflon held in Cyprus in 2016 [85]) and domestic sheep breeds, its descendants should be considered as subspecies, including the domestic sheep [23,86]. This choice would overturn the current scheme classifying the Corsico-Sardinian mouflon as a subspecies of the domestic sheep in databases such as the National Center for Biotechnology Information NCBI (*Ovis aries musimon*. Taxonomy ID 9938) whereas the Cyprus mouflon (*Ovis gmelini ophion*) and the Anatolian mouflon (*Ovis gmelini anatolica*) are classified as subspecies of the wild *Ovis gmelini*. Although it could be objected that domestic breeds are the result of human-mediated selection for more productive traits, the tree topology shows that only some of the mtDNA lineages retrieved were involved in this process. These lineages were pre-existing and not attributable to domestication, and easily distinguishable from each other, in the same way as the evolutionary lineage of the urial is different from those of the other Moufloniformes. In light of these phylogenetic inferences, the scientific name *Ovis aries musimon* to designate the Sardinian and Corsican mouflon (ICZN 2003) should be revised and replaced by *Ovis gmelini musimon* (var. sarda and corsicana, respectively), a name highlighting its descendance from *Ovis gmelini* and in accordance with what proposed by Chalbos et al. [32] for the Corsican mouflon.

The results obtained in the present study constitute an important contribution to the understanding of the matrilineal evolutionary relationships between domestic sheep and wild mouflon, and push toward a prompt revision of the nomenclature of these species. The hope is to definitively bridge the gap between scientists and conservation practitioners and find a meeting point between classic systematics and new molecular evidence.

## Supporting information

S1 Fig Analysis of DNA damage patterns for the three ancient Sardinian samples. (DOCX)

S1 Table Mitogenome sequences dataset. (DOCX)

S2 Table Radiocarbon dating results of the three successfully dated Sardinian samples. (DOCX)

S3 Table Calibration points used for molecular dating. (DOCX)
