# Supplementary figures and images for: Revised phylogeny of mouflon based on expanded sampling of mitogenomes
Source: PLoS One. 2025 May 14;20(5):e0323354. doi: 10.1371/journal.pone.0323354 (PMC12077669; doi:10.1371/journal.pone.0323354)

**S1 Fig**. **Analysis of DNA damage patterns for the three ancient Sardinian samples.**


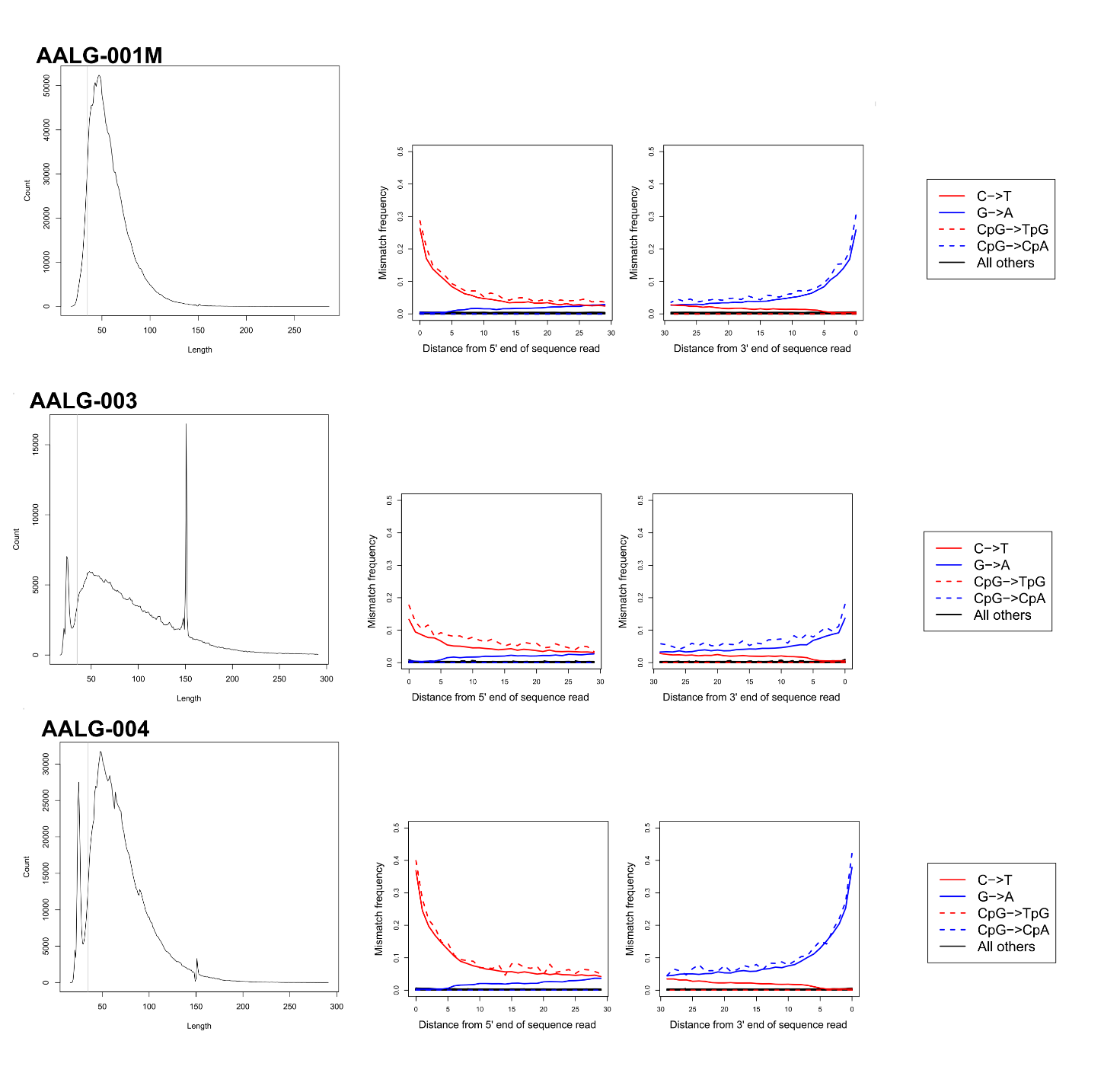

Supplement: S1 Fig — (DOCX) [file pone.0323354.s001.docx]
