# Supplementary material for: Revised phylogeny of mouflon based on expanded sampling of mitogenomes
Source: PLoS One. 2025 May 14;20(5):e0323354. doi: 10.1371/journal.pone.0323354 (PMC12077669; doi:10.1371/journal.pone.0323354)
Supplement: S1 Table — (DOCX) [file pone.0323354.s002.docx]

**S1 Table**. **Mitogenome sequences dataset**

| **Species** | **GenBank Accession** | **Origin** | **Common name, breed & sample type** | **Code** | **Coverage** | **BioProject** | **ENA ID** | **Study Reference** |
| --- | --- | --- | --- | --- | --- | --- | --- | --- |
| *Bos taurus* | NC_006853 | Korea | Domestic cattle |  |  | PRJNA927338 |  |  |
| *Kobus ellipsiprymnus* | NC_020715 | Niger | Waterbuck |  |  | PRJNA927338 |  | Hassanin et al., 2012 |
| *Kobus leche* | NC_018603 | N.A. | Lechwe |  |  | PRJNA927338 |  | N.A. |
| *Redunca arundinum* | NC_020794 | N.A. | southern Reedbuck |  |  | PRJNA927338 |  | Hassanin et al., 2012 |
| *Addax nasomaculatus* | JN632591 | N.A. | Addax |  |  |  |  | Hassanin et al., 2012 |
| *Hippotragus niger* | NC_020713 | N.A. | sable Antelope |  |  | PRJNA927338 |  | Hassanin et al., 2012 |
| *Connochaetes taurinus* | NC_020699 | N.A. | brindled Gnu |  |  | PRJNA927338 |  | Hassanin et al., 2012 |
| *Ovis nivicola* | NC_039431 | Russia | Snow sheep |  |  | PRJNA927338 |  | Dotsev et al., 2019 |
| *Ovis canadensis* | NC_015889 | Canada | Bighorn sheep |  |  | PRJNA927338 |  | Miller et al., 2012 |
| *Ovis ammon* | KT781689 | China | Argali sheep |  |  |  |  | Gan et al., 2016 |
| *Ovis vignei* | NC_026064 | N.A. | Urial sheep |  |  | PRJNA927338 |  | Lv et al., 2015 |
| *Ovis aries* | HM236175 | N.A. | Sheep; breed: Romney; haplogroup A | HPG-A |  |  |  | Meadows et al., 2011 |
| *Ovis aries* | HM236176 | N.A. | Sheep; breed: Karakas; haplogroup B | HPG-B |  |  |  | Meadows et al., 2011 |
| *Ovis aries* | HM236179 | N.A. | Sheep; breed: Morkaraman; haplogroup C | HPG-C |  |  |  | Meadows et al., 2011 |
| *Ovis aries* | HM236181 | N.A. | Sheep; breed: Morkaraman; haplogroup D | HPG-D |  |  |  | Meadows et al., 2011 |
| *Ovis aries* | HM236183 | N.A. | Sheep; breed: Tuj; haplogroup E | HPG-E |  |  |  | Meadows et al., 2011 |
| *Ovis aries* | KF938342 | N.A. | Primitive sheep; breed: Aland | Aland |  |  |  | Lv et al., 2015 |
| *Ovis aries* | KF938355 | N.A. | Primitive sheep; breed: Finnsheep | Finnsheep |  |  |  | Lv et al., 2015 |
| *Ovis aries* | MF004244 | N.A. | Primitive sheep; breed: Hamdani | Hamdani |  |  |  | Mustafa et al., 2017 |
| *Ovis aries* | KF938351 | N.A. | Primitive sheep; breed: Karachai | Karachai |  |  |  | Lv et al., 2015 |
| *Ovis aries* | KF938333 | N.A. | Primitive sheep; breed: Kazakh | Kazakh |  |  |  | Lv et al., 2015 |
| *Ovis aries* | KT148968 | China | Primitive sheep; breed: Oula Tibetan | Oula-Tibetan |  |  |  | Qiao et al., 2020 |
| *Ovis aries* | MT768198 | N.A. | Primitive sheep; breed: Shetland | Shetland |  |  |  | Deng et al., 2020 |
| *Ovis aries* | KF938356 | N.A. | Primitive sheep; breed: Viena | Viena |  |  |  | Lv et al., 2015 |
| *Ovis aries* | N.A. | Iberia | Ancient sample | APOR_002 | 74,71 | PRJEB76447 | ERS20226268 | Morell Miranda, 2023 |
| *Ovis aries* | N.A. | Iberia | Ancient sample | APOR_004 | 24,06 | PRJEB76447 | ERS20226269 | Morell Miranda, 2023 |
| *Ovis aries* | N.A. | Iberia | Ancient sample | APOR_005 | 8,90 | PRJEB76447 | ERS20226270 | Morell Miranda, 2023 |
| *Ovis aries* | N.A. | Iberia | Ancient sample | APOR_008 | 30,09 | PRJEB76447 | ERS20226271 | Morell Miranda, 2023 |
| *Ovis aries* | N.A. | Iberia | Ancient sample | APOR_011 | 65,46 | PRJEB76447 | ERS20226272 | Morell Miranda, 2023 |
| *Ovis aries* | N.A. | Iberia | Ancient sample | APOR_013 | 82,36 | PRJEB76447 | ERS20226273 | Morell Miranda, 2023 |
| *Ovis aries* | N.A. | Sardinia | Ancient sample | ALG-001M | 120,38 | PRJEB76447 | ERS20226274 | This study |
| *Ovis aries* | N.A. | Sardinia | Ancient sample | ALG-003 | 206,83 | PRJEB76447 | ERS20226275 | This study |
| *Ovis aries* | N.A. | Sardinia | Ancient sample | ALG-004 | 113,58 | PRJEB76447 | ERS20226276 | This study |
| *Ovis aries* | N.A. | Anatolia | Ancient sample | tps_062 | 131,10 | PRJEB69690 | ERR12389599, ERR12389600, ERR12389601, ERR12389602, ERR12389603 | Atağ et al. 2024 |
| *Ovis aries* | N.A. | Anatolia | Ancient sample | tps_083 | 142,24 | PRJEB69690 | ERR12389604, ERR12389605, ERR12389606 | Atağ et al. 2024 |
| *Ovis aries* | N.A. | Sardinia | Sheep; breed: Sarda | Sar_s06 | 527,25 | PRJEB76447 | ERS20226277 | This study |
| *Ovis aries* | N.A. | Sardinia | Sheep; breed: Sarda | Sar_s05 | 640,62 | PRJEB76447 | ERS20226278 | This study |
| *Ovis aries* | N.A. | Sardinia | Sheep; breed: Sarda | Sar_s04 | 313,78 | PRJEB76447 | ERS20226279 | This study |
| *Ovis aries* | N.A. | Sardinia | Sheep; breed: Sarda | Sar_s03 | 725,91 | PRJEB76447 | ERS20226280 | This study |
| *Ovis aries* | N.A. | Sardinia | Sheep; breed: Sarda | Sar_s02 | 647,83 | PRJEB76447 | ERS20226281 | This study |
| *Ovis aries* | N.A. | Sardinia | Sheep; breed: Sarda | Sar_s01 | 633,65 | PRJEB76447 | ERS20226282 | This study |
| *Ovis aries* | N.A. | Sardinia | Sheep; breed: Nera di Arbus | Nera_s01 | 849,56 | PRJEB76447 | ERS20226283 | This study |
| *Ovis aries* | N.A. | Sardinia | Sheep breed: Nera di Arbus | Nera_s02 | 539,48 | PRJEB76447 | ERS20226284 | This study |
| *Ovis aries* | N.A. | Sardinia | Sheep; breed: Nera di Arbus | Nera_s03 | 823,00 | PRJEB76447 | ERS20226285 | This study |
| *Ovis aries* | N.A. | Sardinia | Sheep; breed: Nera di Arbus | Nera_s04 | 1277,03 | PRJEB76447 | ERS20226286 | This study |
| *Ovis aries* | N.A. | Sardinia | Sheep; breed: Nera di Arbus | Nera_s05 | 1092,68 | PRJEB76447 | ERS20226287 | This study |
| *Ovis aries* | N.A. | Sardinia | Sheep; breed: Nera di Arbus | Nera_s06 | 1015,40 | PRJEB76447 | ERS20226288 | This study |
| *Ovis gmelini* | N.A. | Iran | Iranian mouflon | Ir_m01 | 451,00 | PRJEB7436 | ERR498073 | Alberto et al., 2018 |
| *Ovis gmelini* | N.A. | Iran | Iranian mouflon | Ir_m02 | 156,00 | PRJEB7436 | ERR466546 | Alberto et al., 2018 |
| *Ovis gmelini* | N.A. | Iran | Iranian mouflon | Ir_m03 | 401,00 | PRJEB7436 | ERR466545 | Alberto et al., 2018 |
| *Ovis gmelini* | N.A. | Iran | Iranian mouflon | Ir_m04 | 286,00 | PRJEB7436 | ERR466544 | Alberto et al., 2018 |
| *Ovis gmelini* | N.A. | Iran | Iranian mouflon | Ir_m05 | 4117,00 | PRJEB7436 | ERR340346 | Alberto et al., 2018 |
| *Ovis gmelini* | N.A. | Iran | Iranian mouflon | Ir_m06 | 1217,00 | PRJEB7436 | ERR332589 | Alberto et al., 2018 |
| *Ovis gmelini* | N.A. | Iran | Iranian mouflon | Ir_m07 | 184,00 | PRJEB7436 | ERR332587 | Alberto et al., 2018 |
| *Ovis gmelini* | N.A. | Iran | Iranian mouflon | Ir_m08 | 644,00 | PRJEB7436 | ERR332582 | Alberto et al., 2018 |
| *Ovis gmelini* | N.A. | Iran | Iranian mouflon | Ir_m09 | 980,00 | PRJEB7436 | ERR332575 | Alberto et al., 2018 |
| *Ovis gmelini* | N.A. | Iran | Iranian mouflon | Ir_m10 | 2704,00 | PRJEB7436 | ERR332573 | Alberto et al., 2018 |
| *Ovis gmelini* | N.A. | Iran | Iranian mouflon | Ir_m11 | 7686,00 | PRJEB7436 | ERR315509 | Alberto et al., 2018 |
| *Ovis gmelini* | N.A. | Iran | Iranian mouflon | Ir_m12 | 2219,00 | PRJEB7436 | ERR157944 | Alberto et al., 2018 |
| *Ovis gmelini* | N.A. | Iran | Iranian mouflon | Ir_m13 | 2330,00 | PRJEB7436 | ERR157942 | Alberto et al., 2018 |
| *Ovis gmelini* | N.A. | Iran | Iranian mouflon | Ir_m14 | 1303,00 | PRJEB7436 | ERR157939 | Alberto et al., 2018 |
| *Ovis gmelini* | N.A. | Iran | Iranian mouflon | Ir_m15 | 8103,00 | PRJEB7436 | ERR157938 | Alberto et al., 2018 |
| *Ovis gmelini* | N.A. | Iran | Iranian mouflon | Ir_m16 | 874,00 | PRJEB7436 | ERR157935 | Alberto et al., 2018 |
| *Ovis gmelini* | N.A. | Iran | Iranian mouflon | Ir_m17 | 666,00 | PRJEB7436 | ERR157932 | Alberto et al., 2018 |
| *Ovis gmelini* | N.A. | Iran | Iranian mouflon | Ir_m18 | 317,00 | PRJEB7436 | ERR157931 | Alberto et al., 2018 |
| *Ovis gmelini* | N.A. | Iran | Iranian mouflon | Ir_m19 | 5064,00 | PRJEB7436 | ERR157930 | Alberto et al., 2018 |
| *Ovis gmelini* | N.A. | Iran | Iranian mouflon | Ir_m20 | 4952,00 | PRJEB7436 | ERR157933 | Alberto et al., 2018 |
| *Ovis gmelini anatolica* | N.A. | Turkey | Anatolian mouflon | OGA_m009 | 57,09 | PRJEB69690 | ERR12389568 | Atağ et al. 2024 |
| *Ovis gmelini anatolica* | N.A. | Turkey | Anatolian mouflon | OGA_m014 | 45,55 | PRJEB69690 | ERR12389569 | Atağ et al. 2024 |
| *Ovis gmelini anatolica* | N.A. | Turkey | Anatolian mouflon | OGA_m018 | 147,35 | PRJEB69690 | ERR12389570, ERR12389571, ERR12389572 | Atağ et al. 2024 |
| *Ovis gmelini anatolica* | N.A. | Turkey | Anatolian mouflon | OGA_m021 | 57,66 | PRJEB69690 | ERR12389573 | Atağ et al. 2024 |
| *Ovis gmelini anatolica* | N.A. | Turkey | Anatolian mouflon | OGA_m022 | 24,10 | PRJEB69690 | ERR12389574 | Atağ et al. 2024 |
| *Ovis gmelini ophion* | N.A. | Cyprus (Paphos Forest) | Cypriot mouflon | Cy_m02 | 1787,44 | PRJEB69690 | ERR12389557 | Atağ et al. 2024 |
| *Ovis gmelini ophion* | N.A. | Cyprus (Paphos Forest) | Cypriot mouflon | Cy_m03 | 395,04 | PRJEB69690 | ERR12389558 | Atağ et al. 2024 |
| *Ovis gmelini ophion* | N.A. | Cyprus (Paphos Forest) | Cypriot mouflon | Cy_m04 | 2456,55 | PRJEB69690 | ERR12389559 | Atağ et al. 2024 |
| *Ovis gmelini ophion* | N.A. | Cyprus (Paphos Forest) | Cypriot mouflon | Cy_m06 | 4853,27 | PRJEB69690 | ERR12389560 | Atağ et al. 2024 |
| *Ovis gmelini ophion* | N.A. | Cyprus (Paphos Forest) | Cypriot mouflon | Cy_m07 | 3048,59 | PRJEB69690 | ERR12389561 | Atağ et al. 2024 |
| *Ovis gmelini ophion* | N.A. | Cyprus (Paphos Forest) | Cypriot mouflon | Cy_m08 | 2470,51 | PRJEB69690 | ERR12389562, ERR12389563 | Atağ et al. 2024 |
| *Ovis gmelini ophion* | N.A. | Cyprus (Paphos Forest) | Cypriot mouflon | Cy_m09 | 4152,50 | PRJEB69690 | ERR12389564 | Atağ et al. 2024 |
| *Ovis gmelini ophion* | N.A. | Cyprus (Paphos Forest) | Cypriot mouflon | Cy_m11 | 172,10 | PRJEB69690 | ERR12389565 | Atağ et al. 2024 |
| *Ovis gmelini ophion* | N.A. | Cyprus (Paphos Forest) | Cypriot mouflon | Cy_m12 | 168,00 | PRJEB69690 | ERR12389566 | Atağ et al. 2024 |
| *Ovis gmelini ophion* | N.A. | Cyprus (Paphos Forest) | Cypriot mouflon | Cy_m13 | 191,60 | PRJEB69690 | ERR12389567 | Atağ et al. 2024 |
| *Ovis gmelini musimon* | N.A. | Corsica (Bavella) | Corsican mouflon | Cor-B_m01 | 177,30 | PRJEB76447 | ERS20226289 | This study |
| *Ovis gmelini musimon* | N.A. | Corsica (Bavella) | Corsican mouflon | Cor-B_m02 | 250,82 | PRJEB76447 | ERS20226290 | This study |
| *Ovis gmelini musimon* | N.A. | Corsica (Bavella) | Corsican mouflon | Cor-B_m03 | 873,98 | PRJEB76447 | ERS20226291 | This study |
| *Ovis gmelini musimon* | N.A. | Corsica (Bavella) | Corsican mouflon | Cor-B_m04 | 514,93 | PRJEB76447 | ERS20226292 | This study |
| *Ovis gmelini musimon* | N.A. | Corsica (Bavella) | Corsican mouflon | Cor-B_m05 | 599,53 | PRJEB76447 | ERS20226293 | This study |
| *Ovis gmelini musimon* | N.A. | Corsica (Bavella) | Corsican mouflon | Cor-B_m06 | 692,80 | PRJEB76447 | ERS20226294 | This study |
| *Ovis gmelini musimon* | N.A. | Corsica (Bavella) | Corsican mouflon | Cor-B_m07 | 553,95 | PRJEB76447 | ERS20226295 | This study |
| *Ovis gmelini musimon* | N.A. | Corsica (Bavella) | Corsican mouflon | Cor-B_m08 | 990,79 | PRJEB76447 | ERS20226296 | This study |
| *Ovis gmelini musimon* | N.A. | Corsica (Bavella) | Corsican mouflon | Cor-B_m09 | 554,32 | PRJEB76447 | ERS20226297 | This study |
| *Ovis gmelini musimon* | N.A. | Corsica (Bavella) | Corsican mouflon | Cor-B_m10 | 880,55 | PRJEB76447 | ERS20226298 | This study |
| *Ovis gmelini musimon* | N.A. | Corsica (Bavella) | Corsican mouflon | Cor-B_m11 | 1389,12 | PRJEB76447 | ERS20226299 | This study |
| *Ovis gmelini musimon* | N.A. | Corsica (Bavella) | Corsican mouflon | Cor-B_m12 | 785,58 | PRJEB76447 | ERS20226300 | This study |
| *Ovis gmelini musimon* | N.A. | Corsica (Monte Cinto) | Corsican mouflon | Cor-C_m01 | 524,36 | PRJEB76447 | ERS20226301 | This study |
| *Ovis gmelini musimon* | N.A. | Corsica (Monte Cinto) | Corsican mouflon | Cor-C_m02 | 162,90 | PRJEB76447 | ERS20226302 | This study |
| *Ovis gmelini musimon* | N.A. | Sardinia (Montes Forest) | Sardinian mouflon | Sar-M_m01 | 893,37 | PRJEB76447 | ERS20226303 | This study |
| *Ovis gmelini musimon* | N.A. | Sardinia (Montes Forest) | Sardinian mouflon | Sar-M_m02 | 273,89 | PRJEB76447 | ERS20226304 | This study |
| *Ovis gmelini musimon* | N.A. | Sardinia (Montes Forest) | Sardinian mouflon | Sar-M_m03 | 29909,29 | PRJEB76447 | ERS20226305 | This study |
| *Ovis gmelini musimon* | MG489885 | Sardinia (Montes Forest) | Sardinian mouflon | Sar-M_m04 |  | PRJEB76447 | ERS20226306 | This study |
| *Ovis gmelini musimon* | N.A. | Sardinia (Montes Forest) | Sardinian mouflon | Sar-M_m05 | 3034,90 | PRJEB76447 | ERS20226307 | This study |
| *Ovis gmelini musimon* | N.A. | Sardinia (Montes Forest) | Sardinian mouflon | Sar-M_m06 | 3412,82 | PRJEB76447 | ERS20226308 | This study |
| *Ovis gmelini musimon* | N.A. | Sardinia (mt. Tonneri) | Sardinian mouflon | Sar-T_m01 | 261,41 | PRJEB76447 | ERS20226309 | This study |
| *Ovis gmelini musimon* | N.A. | Sardinia (mt. Tonneri) | Sardinian mouflon | Sar-T_m02 | 109,35 | PRJEB76447 | ERS20226310 | This study |
| *Ovis gmelini musimon* | N.A. | Sardinia (mt. Tonneri) | Sardinian mouflon | Sar-T_m03 | 208,45 | PRJEB76447 | ERS20226311 | This study |
| *Ovis gmelini musimon* | N.A. | Sardinia (mt. Tonneri) | Sardinian mouflon | Sar-T_m04 | 186,96 | PRJEB76447 | ERS20226312 | This study |
| *Ovis gmelini musimon* | N.A. | Sardinia (mt. Tonneri) | Sardinian mouflon | Sar-T_m06 | 126,51 | PRJEB76447 | ERS20226313 | This study |
| *Ovis gmelini musimon* | N.A. | Sardinia (mt. Tonneri) | Sardinian mouflon | Sar-T_m09 | 67,77 | PRJEB76447 | ERS20226314 | This study |
| *Ovis gmelini musimon* | N.A. | Sardinia (mt. Tonneri) | Sardinian mouflon | Sar-T_m10 | 43,51 | PRJEB76447 | ERS20226315 | This study |
| *Ovis gmelini musimon* | N.A. | Sardinia (mt. Tonneri) | Sardinian mouflon | Sar-T_m11 | 207,65 | PRJEB76447 | ERS20226316 | This study |
| *Ovis gmelini musimon* | N.A. | Sardinia (mt. Tonneri) | Sardinian mouflon | Sar-T_m12 | 4389,10 | PRJEB76447 | ERS20226317 | This study |
| *Ovis gmelini musimon* | N.A. | Sardinia (mt. Tonneri) | Sardinian mouflon | Sar-T_m13 | 184,42 | PRJEB76447 | ERS20226318 | This study |
| *Ovis gmelini musimon* | N.A. | Sardinia (mt. Tonneri) | Sardinian mouflon | Sar-T_m14 | 119,40 | PRJEB76447 | ERS20226319 | This study |
| *Ovis gmelini musimon* | N.A. | Sardinia (mt. Tonneri) | Sardinian mouflon | Sar-T_m15 | 173,17 | PRJEB76447 | ERS20226320 | This study |
| *Ovis gmelini musimon* | N.A. | Sardinia (mt. Tonneri) | Sardinian mouflon | Sar-T_m17 | 251,63 | PRJEB76447 | ERS20226321 | This study |
| *Ovis gmelini musimon* | N.A. | Sardinia (mt. Tonneri) | Sardinian mouflon | Sar-T_m19 | 236,74 | PRJEB76447 | ERS20226322 | This study |
| *Ovis gmelini musimon* | N.A. | Sardinia (mt. Tonneri) | Sardinian mouflon | Sar-T_m20 | 79,86 | PRJEB76447 | ERS20226323 | This study |
| *Ovis gmelini musimon* | HM236184 | Germany | European mouflon | Eur_m |  |  |  | Meadows et al., 2011 |
