# Supplementary material for: Revised phylogeny of mouflon based on expanded sampling of mitogenomes
Source: PLoS One. 2025 May 14;20(5):e0323354. doi: 10.1371/journal.pone.0323354 (PMC12077669; doi:10.1371/journal.pone.0323354)
Supplement: S2 Table — (DOCX) [file pone.0323354.s003.docx]

| **Sample ID** | **Radiocarbon date (uncalibrated ± 1 SD, before present*)** | **Radiocarbon date (calibrated, AD, 95.4% probability)** | **Accession** |
| --- | --- | --- | --- |
| ALG-001M | 1716 ± 28 | 252 - 292 | Ua-78301 |
| ALG-003 | 208 ± 47 | 1528 - 1949 | Ua-78302 |
| ALG-004 | 1606 ± 61 | 262 - 593 | Ua-78303 |

**S2 Table**. **Radiocarbon dating results of the three successfully dated Sardinian samples.**

* Present = 1950 for radiocarbon dating
