# Supplementary material for: Revised phylogeny of mouflon based on expanded sampling of mitogenomes
Source: PLoS One. 2025 May 14;20(5):e0323354. doi: 10.1371/journal.pone.0323354 (PMC12077669; doi:10.1371/journal.pone.0323354)
Supplement: S3 Table — (DOCX) [file pone.0323354.s004.docx]

**S3 Table**. **Calibration points used for molecular dating.**

|  | **CP** | **95% C.I.** | **Mean** | **ST.Err** | **Reference study** |
| --- | --- | --- | --- | --- | --- |
| 1 | Kobus | 2000000 - 3000000 | 2500000 | 707107 | # |
| 2 | Crown Hippotragini | 3600000 - 6500000 | 5050000 | 2050610 |  |
| 3 | Crown Reduncini | 5100000 - 7000000 | 6050000 | 1343503 |  |
| 4 | Stem Hippotragini | 6400000 - 13000000 | 9700000 | 4666905 |  |
| 5 | tps083 |  | 8314 | 102 | **§** |
| 6 | tps062 |  | 8796 | 188 |  |
| 7 | ALG01 |  | 1716 | 28 | ***** |
| 8 | APOR05 |  | 4946 | 95 | **°** |
| 9 | APOR08 |  | 7147 | 123 |  |
| 10 | APOR11 |  | 5459 | 128 |  |

#Bibi et al. 2013; § Yurtman et al. 2021; °Morell-Miranda 2023; *This study.
